# Depression, neuroticism and 2D:4D ratio: evidence from a large, representative sample

**DOI:** 10.1038/s41598-020-67882-x

**Published:** 2020-07-07

**Authors:** Leopold Maria Lautenbacher, Levent Neyse

**Affiliations:** 1 SOEP, German Institute for Economic Research (DIW), Mohrenstrasse 58, 10117 Berlin, Germany; 2 Department of Psychology, Free University, Berlin, Germany

**Keywords:** Psychology, Predictive markers, Endocrinology

## Abstract

A body of literature reports higher rates of depression and neuroticism in female samples compared to male samples. Numerous studies have investigated the role of prenatal sex hormone exposure in this sex difference, using the ratio between the second and fourth digit of the hand (“2D:4D”) as a putative marker. However, the sample sizes of those studies were mostly small and results remained inconclusive. The aim of the present study is to test the suggested associations between depression, neuroticism and the 2D:4D ratio in a large, representative sample of over 3,000 German individuals. It was hypothesized that a higher 2D:4D (supposedly representing a more “feminine” prenatal hormone exposure) would positively predict (1) one’s history of depression as well as (2) neuroticism rates and (3) acute depressive symptom scores. Controlling for biological sex, we only found suggestive evidence for linear associations with neuroticism in the case of left hand 2D:4D ratios and the mean 2D:4D of both hands. However, additional analyses indicated that these results may have been spurious due to confounding. Our findings suggest that the 2D:4D ratio is not a relevant predictor of depression, while there was mixed evidence in the case of neuroticism.

## Introduction

Depressive disorders are among the leading causes of years lived with disability in the world^1^. Among countless negative effects on health, depression significantly impairs psycho-social functioning^2^ and may be a risk factor for other mental disorders such as dementia^3^. It is also a predictor of negative physical health outcomes such as coronary heart disease^4^ and obesity^5^. Given their consequences, it is unsurprising that mood disorders (including depressive disorders) cause the highest societal costs of all mental disorders in the European Union^6^. Of similar importance to public health is the personality construct *neuroticism*, a factor in the five-factor model of personality which is characterized by negative affect and the disturbed thoughts and behaviors associated with emotional distress^7^. Neuroticism has been related to depression and other mental disorders in multiple reviews and meta-analyses^8–10^. It is likely that several mechanisms explain the association between neuroticism and mental illness. For example, the association may partly reflect shared roots and conceptual overlap between constructs^9^. Furthermore, the vulnerability model proposes that personality traits can contribute to the development of disorders and that one may use personality scores to predict the likelihood of developing certain conditions in the future^9^. In fact, neuroticism has been labeled as an efficient marker of risk for developing psychopathology^11^. While neuroticism has often been used as a predictor of other outcomes, less is known about the causes of individual differences in neuroticism. Consequently, there is much to gain from investigating how depressed or neurotic individuals differ from non-depressed or less neurotic individuals, as this may improve our understanding of the etiology of depression and mental disorders in general.

One common observation about depression and neuroticism is the sex difference in their prevalence. Most studies have found higher rates of depression among female participants compared to male participants^12–15^. A recent meta-analysis indicated that this observation holds true across different nations and estimated an odds ratio of 1.95 for gender differences in diagnoses of major depression^16^. That is, the proportion of individuals with a diagnosis of major depression was approximately twice as large in female samples compared with male samples, on average. Similarly, many studies show that female participants tend to score higher on neuroticism scales than male participants^17–19^. The aforementioned reviews suggest various explanations for the sex difference in depression rates, which show that sex differences in reporting biases cannot be the main reason. Some examples of these explanations include social and economic discrimination against women, conflicts caused by traditional gender roles, hormonal fluctuations, a more ruminative style of responding to depressed moods that could lead to higher chronicity and the effects of pubertal hormonal changes on neurotransmitter activity^12–15^. As none of these can fully explain the sex difference in the findings on its own, it appears likely that a combination of factors contributes to the sex difference. However, there may also be other variables involved that have not yet been fully explored.

One relatively recent line of research trying to explain sex differences in human behavior investigates possible associations of behaviors with one’s prenatal exposure to testosterone and estrogen. During gestation, the fetus’s brain receptors are exposed to sex hormones, which affect the organizational development of the brain and may thus influence behavior later in life^20^. This exposure is thought to be related to the differentiation of the genitals which in turn is determined by genes of the *Hox* family. Significantly, the same genes have also been implicated as a requirement for the growth and patterning of the digits^21^. For this reason, finger length may be correlated with prenatal testosterone and estrogen exposure. In particular, the ratio between the second and fourth digits of the hand (2D:4D hereafter) has been studied for use as a proxy measure of prenatal hormone exposure ever since significantly lower 2D:4D values were observed in male children compared to female children and adults^22^. A lower 2D:4D, which is more common in men, is taken to indicate a relatively higher prenatal exposure to testosterone and a relatively lower prenatal exposure to estrogen. While the sex difference in 2D:4D ratios is a well-established finding^23^, evidence for the appropriateness of 2D:4D ratios as a proxy measure of prenatal hormone exposure is limited: experimental studies on rodents^24–26^, correlational evidence from non-human primate research^27^ and some studies on individuals with sex-hormone-related syndromes^28,29^ support the claimed link, but the direct evidence from studies that analyzed amniotic fluid is less clear than expected^30,31^. In support of its use as a proxy measure, it has been argued that 2D:4D ratios are easier to obtain than direct measurements of prenatal sex hormone exposure and may reflect the pooled effect of sex hormone exposure throughout gestation, thus being more resistant to daily variations than a single direct measurement^32^. However, there is also evidence indicating that the association with prenatal hormones may not be strong enough for 2D:4D to be a robust marker^33–35^. Despite conflicting opinions about its usefulness, various studies have employed 2D:4D as a proxy measure for prenatal sex hormone exposure in order to explain sexually dimorphic behaviors, including depression and neuroticism.

At least one plausible mechanism has been proposed in the literature for the effect of prenatal sex hormone exposure on the development of depression and neuroticism: Higher prenatal testosterone exposure has been associated with increased activation to positive stimuli^36^, while depression and neuroticism are associated with increased activation to negative stimuli^37,38^. Therefore, higher prenatal testosterone exposure may act as a protective factor against depression later in life by influencing approach-avoidance tendencies and reward system sensitivity. We were able to identify 16 previous papers that analyzed 2D:4D in the context of depression or neuroticism. One could expect there to be a positive association between the two constructs and 2D:4D, as a higher 2D:4D ratio supposedly represents a *more feminine* hormone profile during gestation. However, the research so far has not yielded conclusive results. In the case of depression, some studies reported null results^39–44^, while others found significant positive as well as significant negative associations with 2D:4D. Moreover, all significant results were restricted to either female or male participant groups, with no study reporting a significant relationship between depression and 2D:4D in both sex groups: There was one significant positive^45^ and one significant negative^46^ association in females, as well as two significant positive^47,48^ and two significant negative^49,50^ associations in males. In the case of neuroticism, four out of five papers found significant positive associations^40,51–53^, with the fifth paper reporting nonsignificant results^54^. However, as was the case with depression, most of the significant results were restricted to either female or male participants (two positive associations in females, one positive association in males), while the results of Austin and colleagues^40^ were only statistically significant for the combined sample. The majority of effect sizes were small for both depression and neuroticism ($r < 0.3$). The inconclusiveness in the literature may have been exacerbated by the fact that most of the previous studies had moderately small sample sizes (for depression: mean $n = 227$, median $n = 196$; for neuroticism: mean $n = 561$, median $n = 265$) and mainly included university or college students as participants. A detailed tabular summary of these previous findings can be found in the first section of the supplementary materials.

The aim of the present study is to bring some clarity to the discussion by assessing the supposed associations between 2D:4D and depression as well as 2D:4D and neuroticism in a large and representative sample, using interviewer-measured 2D:4D data. In 2018, the 2D:4D ratios of over 3,000 individuals were measured in the Innovation Sample of the German Socio-Economic Panel Study^55^, an ongoing longitudinal and nationally representative study in Germany. The same sample also collected data on participants’ history of depression, neuroticism scores and acute depressive symptom scores. Given these measures as well as the theoretical background of 2D:4D research and the sex difference in depression and neuroticism, we evaluated the following three hypotheses using regression analyses with 2D:4D and biological sex as the predictor variables:

While controlling for biological sex, a higher (more “feminine”) 2D:4D predicts:

1. having been diagnosed with a depressive disorder by a medical professional,
2. higher levels of neuroticism,
3. higher levels of acute depressive symptoms.

To the best of our knowledge, this study is the largest to date to investigate 2D:4D in the context of depression and has ample power to detect relevant associations. Furthermore, while the largest study on 2D:4D and neuroticism to date (by amount of data gathered) calculated national 2D:4D mean values from individual self-measured 2D:4D data to analyze national trends^52^, the present study uses interviewer-measured 2D:4D data and is also able to provide insight into between-individual variation.

## Results

Three outcome variables were assessed: a dichotomous self-report indicating whether the participant had received a diagnosis of a depressive disorder by a medical professional in the past (“History of depression”), neuroticism scores on an abbreviated five-factor personality questionnaire (Big Five Inventory-SOEP)^56^ and acute depressive symptom scores measured by the Patient Health Questionnaire-2 (PHQ-2)^57,58^. More information about these measures is reported in the methods section. All pairwise correlation coefficients for the variables analyzed in our study, including both predictor and outcome variables, are presented in Table 1. No correlation coefficient was estimated for the relationship between the two binary variables (biological sex and history of depression). Almost all of the correlations had corresponding *p* values below 0.05, except the associations between right hand 2D:4D and history of depression, between age and biological sex and between age and history of depression.

**Table 1:** Pairwise correlations between the variables studied.

Variable	R2D:4D	L2D:4D	M2D:4D	AGE	FEM	HIST	NEUR	PHQ-2
R2D:4D	1							
L2D:4D	0.34	1						
M2D:4D	0.80	0.84	1					
AGE	− 0.04	− 0.05	− 0.06	1				
FEM	0.04	0.07	0.06	< − 0.01	1			
HIST	0.02	0.04	0.04	− 0.02	−	1		
NEUR	0.04	0.05	0.05	− 0.09	0.24	0.21	1	
PHQ-2	0.05	<− 0.01	0.03	− 0.13	0.01	0.25	0.28	1

2D:4D = the length of the second digit divided by the length of the fourth digit of the hand (R $=$ right hand, L $=$ left hand, M $=$ mean of both hands), FEM = female sex, i.e., a value of 1 on our biological sex variable, HIST = history of depression, NEUR $=$ neuroticism, measured using the Big Five Inventory-SOEP, PHQ-2 $=$ Patient Health Questionnaire-2, a measure of acute depressive symptoms. $p < 0.05$ for all coefficients except for the associations between R2D:4D and HIST, between AGE and FEM and between AGE and HIST.

Descriptive statistics and the results of tests comparing female and male participants on the variables for which sex differences were expected are presented in Table 2. Replicating previous findings, there were significant differences ($p < 0.005$) between female and male participants regarding 2D:4D, history of depression and neuroticism, the latter being particularly pronounced ($d = -0.51$). The direction of these effects was also as expected: female participants had higher 2D:4D, were more likely to report having been diagnosed with depression and had higher neuroticism scores. While the odds ratio for the sex difference in history of depression was very similar to the estimate reported in a previous meta-analysis^16^, no relevant sex difference was found for acute depressive symptoms measured by the PHQ-2. The size of the sex difference in 2D:4D values in this sample was particularly small compared to the typical range ($0.09< d < 0.61$) based on a previous meta-analysis^23.^

**Table 2:** Descriptive statistics and results of tests comparing female and male participants.

Variable	*n*	*M*	$SD$	Cases	Controls	*t* / $\chi ^2$	*d*/*OR*	*p*
R2D:4D						− 2.46	− 0.08	0.01
Female	1,834	1.001	0.049					
Male	1,555	0.997	0.047					
L2D:4D						− 3.92	− 0.14	< 0.0001
Female	1,827	1.003	0.055					
Male	1,560	0.996	0.054					
HIST						23.78	1.99	< 0.0001
Female	1,834			168	1,666			
Male	1,555			75	1,480			
NEUR						− 14.70	− 0.51	< 0.0001
Female	1,794	4.264	3.711					
Male	1,522	2.382	3.630					
PHQ-2						0.47	0.027	0.64
Female	647	2.989	1.239					
Male	563	3.023	1.287					

2D:4D $=$ the length of the second digit divided by the length of the fourth digit of the hand (R $=$ right hand, L $=$ left hand), $M=\text {mean}$, HIST = history of depression, NEUR $=$ neuroticism, $OR =$ odds ratio, PHQ-2 $=$ Patient Health Questionnaire-2, $SD =\text {standard deviation}$. HIST was measured as a binary variable (cases = diagnosed at least once, controls $=$ never diagnosed). $\chi ^2$ and *OR* are only reported for HIST.

Logistic regression was performed for history of depression, while linear regression models were used for neuroticism and PHQ-2 scores. Our main analyses included 2D:4D and biological sex as predictors. As previous studies found different sex-specific associations, we also conducted exploratory analyses including an interaction term between 2D:4D and biological sex. The results for right hand 2D:4D, left hand 2D:4D and the mean 2D:4D of both hands are shown in Tables 3, 4 and 5, respectively. Note that odds ratios are displayed for the binary outcome (history of depression), while standardized linear regression coefficients are shown in the case of neuroticism and PHQ-2. The choice of models reported in this article was guided by the pre-registered analysis plan of a related study (https://osf.io/9pw72/).

No association with right hand 2D:4D reached our thresholds for suggestive or significant evidence ($p < 0.05$ and $p < 0.005$, respectively) in both main and exploratory analyses. Regarding left hand 2D:4D, only the association with neuroticism in the exploratory analysis had a *p*-value below our threshold for suggestive evidence ($\beta = 0.05$, $p = 0.047$). Similarly, there was suggestive evidence for an association with neuroticism, but not with the depression measures, in the main and exploratory analyses of the mean 2D:4D ratio of both hands ($\beta = 0.04$, $p = 0.03$ and $\beta = 0.05$, $p = 0.046$, respectively). However, we also conducted additional exploratory analyses in which age was controlled for or in which 2D:4D observations were grouped by interviewers (reported in the third and sixth section of the supplementary materials, respectively). Controlling for age or grouping by interviewers both resulted in *p* values above the cut-off of 0.05 for these associations with neuroticism; the suggested relationship may therefore be spurious. There was no suggestive or significant evidence for any interaction effect with biological sex. All effect sizes for 2D:4D were very small, ranging from $\beta = -0.01$ to $\beta = 0.05$ for the point estimates in the linear regression models. Additional analyses including quadratic terms for 2D:4D yielded similar effect sizes and are reported in the fourth section of the supplementary materials. It should be acknowledged that the results for the quadratic term of right hand 2D:4D also reached our threshold for suggestive evidence (HIST: $p = 0.03$; NEUR: $p = 0.04$; PHQ-2: $p = 0.048$).

**Table 3:** Main and exploratory regression analyses using the right hand 2D:4D ratio.

Variable	HIST ($n = 3{,}389$)		NEUR ($n = 3{,}316$)		PHQ-2 ($n = 1{,}210$)	
	*OR* [CI]	*p*	$\beta$ [CI]	*p*	$\beta$ [CI]	*p*
R2D:4D	1.08 [0.95, 1.23]	0.23	0.03 [− 0.01, 0.06]	0.13	0.05 [− 0.01, 0.10]	0.08
FEM	1.98 [1.49, 2.62]	<0.001	0.49 [0.43, 0.56]	<0.001	− 0.03 [− 0.15, 0.08]	0.59
**Interaction test**						
R2D:4D	1.05 [0.83, 1.34]	0.66	0.03 [− 0.02, 0.08]	0.31	0.04 [− 0.04, 0.12]	0.34
FEM	1.97 [1.49, 2.62]	<0.001	0.49 [0.43, 0.56]	<0.001	− 0.03 [− 0.14, 0.08]	0.61
R2D:4D × FEM	1.04 [0.78, 1.38]	0.79	<− 0.01 [− 0.07, 0.07]	0.98	0.02 [− 0.09, 0.13]	0.78

CI $=$ 95% confidence interval, FEM $=$ female sex, HIST $=$ history of depression, NEUR $=$ neuroticism, $OR =$ odds ratio, PHQ-2 $=$ Patient Health Questionnaire-2, R2D:4D $=$ the length of the second digit divided by the length of the fourth digit of the right hand. $\beta$ indicates the change in the *z*-standardized outcome variables for a change of one standard deviation in 2D:4D while controlling for biological sex or the change in the case of female instead of male participants while controlling for 2D:4D.

**Table 4:** Main and exploratory regression analyses using the left hand 2D:4D ratio.

Variable	HIST ($n = 3{,}387$)		NEUR ($n = 3{,}313$)		PHQ-2 ($n = 1{,}204$)	
	*OR* [CI]	*p*	$\beta$ [CI]	*p*	$\beta$ [CI]	*p*
L2D:4D	1.13 [0.99, 1.29]	0.07	0.03 [<− 0.01, 0.07]	0.051	0.01 [− 0.05, 0.07]	0.78
FEM	1.97 [1.49, 2.62]	<0.001	0.49 [0.42, 0.56]	<0.001	0.02 [− 0.14, 0.09]	0.70
**Interaction test**						
L2D:4D	1.08 [0.85, 1.37]	0.53	0.05 [<0.01, 0.10]	0.047	− 0.01 [− 0.10, 0.08]	0.89
FEM	1.97 [1.48, 2.61]	<0.001	0.49 [0.42, 0.56]	<0.001	− 0.02 [− 0.14, 0.09]	0.71
L2D:4D x FEM	1.07 [0.81, 1.42]	0.63	− 0.03 [− 0.10, 0.04]	0.40	0.03 [− 0.09, 0.14]	0.67

CI $=$ 95% confidence interval, FEM $=$ female sex, HIST $=$ history of depression, L2D:4D $=$ the length of the second digit divided by the length of the fourth digit of the left hand, NEUR $=$ neuroticism, $OR =$ odds ratio, PHQ-2 $=$ Patient Health Questionnaire-2. $\beta$ indicates the change in the *z*-standardized outcome variables for a change of one standard deviation in 2D:4D while controlling for biological sex or the change in the case of female instead of male participants while controlling for 2D:4D.

**Table 5:** Main and exploratory regression analyses using the mean 2D:4D ratio of both hands.

Variable	HIST ($n = 3{,}337$)		NEUR ($n = 3{,}264$)		PHQ-2 ($n = 1{,}185$)	
	*OR* [CI]	*p*	$\beta$ [CI]	*p*	$\beta$ [CI]	*p*
M2D:4D	1.14 [0.99, 1.30]	0.06	0.04 [<0.01, 0.07]	0.03	0.03 [− 0.02, 0.09]	0.24
FEM	1.95 [1.46, 2.59]	<0.001	0.49 [0.42, 0.55]	<0.001	− 0.03 [− 0.15, 0.08]	0.56
**Interaction test**						
M2D:4D	1.08 [0.85, 1.37]	0.52	0.05 [<0.01, 0.10]	0.046	0.02 [− 0.07, 0.10]	0.71
FEM	1.94 [1.45, 2.58]	<0.001	0.49 [0.42, 0.55]	<0.001	− 0.03 [− 0.15, 0.08]	0.57
M2D:4D x FEM	1.08 [0.81, 1.44]	0.62	− 0.02 [− 0.09, 0.05]	0.54	0.03 [− 0.08, 0.14]	0.54

CI $=$ 95% confidence interval, FEM $=$ female sex, HIST $=$ history of depression, M2D:4D $=$ the mean of the digit ratios of the right hand and left hand, NEUR $=$ neuroticism, $OR =$ odds ratio, PHQ-2 $=$ Patient Health Questionnaire-2. $\beta$ indicates the change in the *z*-standardized outcome variables for a change of one standard deviation in 2D:4D while controlling for biological sex or the change in the case of female instead of male participants while controlling for 2D:4D.

## Discussion

A body of literature suggests associations between 2D:4D, depression and neuroticism. However, the results are inconclusive and sex-specific. We have tested the relationships using the largest dataset to this date on this subject.

We found no convincing evidence for a linear association of 2D:4D with depression, while there was mixed evidence in the case of neuroticism. Our first and third hypotheses could not be supported. Regarding the second hypothesis (concerning neuroticism), suggestive evidence was found in the analyses for left hand 2D:4D and mean 2D:4D reported in this article. However, the fact that controlling for age or for systematic measurement differences between interviewers in additional analyses both resulted in $p > 0.05$ for all 2D:4D associations (reported in the supplementary materials) casts serious doubt on whether the second hypothesis can be considered supported. While our findings are in line with previous studies that reported null results, it should also be noted that no clear trend favoring one sex or a certain direction of effect is apparent in the literature. We did not find any convincing evidence for sex-specific effects as well. The various positive and negative associations previously observed may have been related to “researcher degrees of freedom”, to measurement differences and to the samples used, most of which were small and consisted of university students. However, even restricting our sample to participants below the age of 30 did not affect our results in meaningful ways (details are reported in the supplementary materials).

There are plausible explanations for the null results presented. For one, the effect of prenatal sex hormone exposure may be miniscule, making sample size requirements for adequate statistical power even higher, or may only be observed when additional confounding factors are accounted for, if there is an effect at all. Furthermore, as our additional exploratory analyses using quadratic terms (reported in the supplementary materials) suggested that there may be nonlinear associations between right hand 2D:4D and our outcome measures, the relationship between 2D:4D and depression or neuroticism may be more complex than previously theorized. However, if these results were replicated, it would still be unclear how the observation of a nonlinear relationship with 2D:4D can help explain the observed sex differences in depression and neuroticism.

Finally, the null results could be explained by a lack of association between 2D:4D and prenatal sex hormone exposure. While the theory behind 2D:4D research may appear sound, strong evidence supporting its appropriateness as a proxy variable is lacking. Some findings indicate that other factors contribute to the variation in 2D:4D more strongly than prenatal testosterone does^33–35^. For example, Berenbaum and colleagues^33^ found no significant differences in mean 2D:4D ratios and 2D:4D variability between women with functioning androgen receptors and women with complete androgen insensitivity. Researchers who experimentally manipulated prenatal testosterone exposure in non-human primates also concluded that various factors apart from the level of sex hormone exposure influence how 2D:4D manifests after birth^59^. Further adding to the uncertainty surrounding 2D:4D, a genome-wide association study with over 15,000 participants was unable to find strong genetic evidence to support the use of 2D:4D as a biomarker of prenatal testosterone exposure^60^. The direct evidence from two analyses involving the amniotic fluid is limited as well. While Lutchmaya and colleagues^30^ only found a significant association in the right hand, Ventura et al.^31^ reported significant associations only for females. However, it has also been argued that amniotic fluid data may not be optimal for assessing the relationship between 2D:4D and sex hormones because 2D:4D may already be sexually dimorphic by the end of the first trimester of pregnancy, while amniotic fluid is typically sampled later during gestation for safety reasons^61^. In support of the validity of 2D:4D, a relationship with prenatal sex hormone exposure has been demonstrated in animal experiments^24–26^ and has been observed in non-human primates^27^ as well as individuals with congenital adrenal hyperplasia, a condition exposing fetuses to high levels of androgens before birth^28,29^. It has also been argued that even an imperfect proxy measure could be informative given a large enough sample^62^. Still, as long as we are unable to explain a reasonably high proportion of the variation in 2D:4D and control for confounding factors, it may be unwise to interpret 2D:4D as a proxy variable. Further discussions on the topic have recently been published elsewhere^63,64^. Additional research on the development of 2D:4D could improve our understanding of previous findings linking 2D:4D with psychological constructs.

The current study has various strengths. For one, 2D:4D ratios were measured directly by interviewers using calipers. By doing so, we were able to avoid both the distortion of soft tissue that can occur with indirect measurements such as photocopies^23,65^ as well as the higher inaccuracy present in participant-measured 2D:4D data^52^. We also accounted for possible systematic differences in 2D:4D measurements between individual interviewers using multilevel modeling (reported in the supplementary materials). Furthermore, validated measures of depression and neuroticism were used. Most importantly, the analyses were performed on a large, representative sample which provided high statistical power and external validity. However, there are also several limitations. First of all, the hypotheses and the exact analysis plan were not pre-registered. While analytical decisions were informed by the pre-registration of a related study, the influence of “researcher degrees of freedom” still has to be expected for our results as well. Moreover, we used abbreviated self-report scales and the item measuring the history of depression has not been fully validated. The number of participants with a history of depression could have been underestimated. Structured clinical interviews or longer questionnaires might have produced more accurate measurements. The validity of our results may also be restricted to European populations, due to ethnic and cultural differences in the analyzed variables. Finally, we used a binary framework of sex, ignoring the implications of intersexuality and gender identity. Data that is more detailed in this regard could provide more nuanced insight.

## Conclusion

There was neither suggestive nor significant evidence for a linear association between the 2D:4D ratio and depression in a representative sample of over 3,00 German individuals. Mixed evidence was found for an association with neuroticism and there was suggestive evidence for possible nonlinear relationships between 2D:4D and depression and neuroticism. Future research efforts should consider focusing on the causes of variation in 2D:4D instead of studying associations with behavior later in life, as the 2D:4D ratio’s appropriateness for indicating prenatal sex hormone exposure seems questionable.

## Methods

### Sample

We used data from the Innovation Sample of the German Socio-Economic Panel Study (SOEP-IS)^55^ up to the survey year 2018. In 2018, 3,509 of 3,958 individuals consented to having their 2D:4D ratios measured. Of these 3,509 participants, 40 participants were excluded from all analyses due to digit or hand injuries. 12 were excluded from analyses on the right hand 2D:4D due extreme 2D:4D data indicating substantial measurement error, i.e., a ratio smaller than 0.8 or larger than 1.2. 32 participants were excluded from analyses on the left hand for the same reason. The exclusion groups overlapped only for one participant, who was excluded from both.

Combined data on right hand 2D:4D and history of depression was available for 1,834 women and 1,555 men (3,389 participants in total), with an age range between 18 and 98 years ($M = 55, SD = 18$). For 3,316 of these participants, combined data on right hand 2D:4D and neuroticism was available as well. In a subset of the larger sample, 647 women and 563 men (1,210 participants in total) had also provided data on acute depressive symptoms.

Ethical permission was provided by the Scientific Advisory Board of DIW Berlin and informed consent was obtained from all participants included in the analyses. On the variables we studied, there were no significant differences between participants who consented to getting their 2D:4D measured and those who declined consent. The corresponding tests are reported in the second section of the supplementary materials.

Complete case analyses were used because the main missingness mechanisms (determining which participants in the SOEP-IS [1] were assessed on the measures of interest and; [2] had reasonably accurate 2D:4D data) were estimated to be unrelated to the outcome variables or possible confounders. Therefore, the risk of bias from using complete case analyses compared to imputation methods was deemed negligible^66^.

### Measures

#### 2D:4D measurement

The data collection was performed by 263 trained interviewers in SOEP-IS 2018 Wave between September 2018 and December 2018. 2D:4D measurement was a part of the module entitled *2D:4D and Economic Preferences in a Large, Representative Sample of Germans* by the second author of the current study (LN), Anna Dreber and Magnus Johannesson. The study was pre-registered at https://osf.io/9pw72/. Lengths of index (2D) and ring fingers (4D) from both hands were measured with digital calipers. 2D:4D ratios were calculated by dividing the length of the index finger to the length of the ring finger. There were two reasons for using digital calipers instead of flatbed scanners. First, collecting 2D:4D data through hand scans is more problematic in the field as images involve sensitive data (i.e., fingerprints). Second, digital scanners are less mobile than digital calipers. The full measurement protocol can be found at the OSF repository under the link https://osf.io/9q8gp/. Prior to data collection, the protocol was cross-tested with research assistants and LN multiple times. The measurements done with the protocol and digital calipers were almost identical to those done with flatbed scanners and image editing software (GIMP).

#### Depression measures

Two variables measuring depression were used in the analyses, one relating to diagnostic history and one to acute symptoms.

For assessing the history of depression, a number of participants were asked whether they had ever received a clinical diagnosis of a depressive disorder from a medical professional ($n = 1{,}649$). Other participants were posed a similar question but their responses were restricted to the past two years instead of their lifetime ($n = 1{,}740$), due to survey design changes in SOEP-IS unrelated to this study. However, the second question was also asked several times between 2013 and 2018, thereby covering a span of 7 years. These two questions were combined into one binary variable. If a participant answered “Yes” at least once in any of the included survey years (2009–2018), they received a value of 1 on the variable. Otherwise, they received a value of 0, indicating no known history of depression. To ensure the validity of this variable, participants with and without a known history of depression were compared on neuroticism and acute depressive symptom scores. Significant large effects in the expected directions were observed ($d = -0.80$ and $d = -1.08$, respectively; $p < 0.005$), supporting the validity. Furthermore, there were no suggestive or signifiant differences in neuroticism ($p = 0.78$) and acute depressive symptoms ($p = 0.89$) between the two question groups (“ever” vs. “past two years”), supporting the combination of these questions.

For assessing acute depressive symptoms, responses on a well-validated two-item scale, the Patient Health Questionnaire-2^57, 58^, was used. The two items of the PHQ-2 assess how often respondents have been bothered by any of the following problems over the past two weeks: (1) little interest or pleasure in doing things and (2) feeling down, depressed, or hopeless. Responses can range from 1 (“Not at all”) to 4 (“Nearly every day”). These two problem domains correspond to the two main diagnostic criteria of major depressive disorder according to the *Diagnostic and Statistical Manual of Mental Disorders* , Fifth Edition.^67^. For the analysis, a score obtained by the sum of the two variables was used, ranging from 2 to 8. Each participant completed the PHQ-2 only once, which is why no selection or summary across survey years had to be made.

#### Neuroticism measure

Neuroticism was assessed using three items of an abbreviated five-factor personality questionnaire, the Big Five Inventory-SOEP^56^. Its validity and reliability compared to the standard NEO-PI-R^68^ were analyzed in the same German panel study and have been found to be acceptable, with a convergent correlation of .86 with the neuroticism scale in the NEO-PI-R and a test-retest correlation of .74 for neuroticism after 18 months^69^. Neuroticism scores were available for several points in time between 2005 and 2017. For each participant, the neuroticism scores from the time of measurement closest to the time of the 2D:4D measurement were used. Respondents were asked to rate whether they are someone who (1) “worries a lot”, (2) “is somewhat nervous” and (3) “deals well with stress” on a range from 1 (“Does not apply at all”) to 7 (“Fully applies”). These three items were then summed up to obtain a score for neuroticism, ranging from − 5 to 13 because the third item assessing dealing with stress was inverted for the addition.

### Statistical analysis

We computed pairwise correlations to assess the baseline associations of the variables involved in our study. As we were interested in the role of the 2D:4D ratio in explaining sexually dimorphic behavior, the assumption of sex differences in our data was evaluated by comparing female and male participants on the relevant variables using two-tailed t-tests. Pearson’s chi-squared test ($\chi ^2$) was used in the case of history of depression.

For evaluating our hypotheses, we used logistic regression in the case of history of depression and linear regression for neuroticism and acute symptom scores. Regression analyses were performed separately for each combination of one of the 2D:4D measures (right hand 2D:4D, left hand 2D:4D and mean 2D:4D of both hands) with one of the outcome variables. For all analyses, the 2D:4D variables as well as neuroticism and acute depressive symptom scores were standardized to yield more interpretable results. Biological sex and history of depression as binary variables remained unstandardized. On the biological sex variable, males were assigned a value of 0 and females a value of 1. Both main and exploratory regression analyses were performed: The main analyses included 2D:4D and biological sex as the predictors, while the exploratory analyses also included an interaction term to account for the possibility of an interaction effect between 2D:4D and biological sex. To aid interpretation, the interaction in the logistic model reported as an odds ratio is illustrated in the supplementary materials.

In this article, we report the results of the main and exploratory analyses. The supplementary materials include additional analyses controlling for age because we observed small but consistent correlations of age with all 2D:4D measures and two of the outcome measures. Previous studies also reported lower neuroticism scores in older adults^70,71^. We also performed additional analyses restricted to participants below the age of 30 (reported in the supplementary materials). Some previous studies suggested nonlinear relationships between 2D:4D and personality traits such as altruism^72,73^ and between testosterone and depression^74^, which is why additional analyses including quadratic terms of right hand and left hand 2D:4D are also reported and illustrated in the supplementary materials. To investigate the influence of systematic differences between interviewers, multilevel modeling was performed, grouping 2D:4D observations by interviewers (reported in the supplementary materials). To further inspect our 2D:4D data for anomalies, digit asymmetry measures were analyzed (reported in the supplementary materials). We also tested for an interaction between 2D:4D and history of depression (reported in the supplementary materials).

The choice of the regression models reported in this article was guided by the pre-registered analysis plan of the aforementioned study for SOEP-IS digit ratio data and economic preferences (https://osf.io/9pw72/). Following the guidance of the pre-registered analysis plan, results with *p* values between 0.05 and 0.005 were considered “suggestive evidence”, while results with *p* values below 0.005 were considered “significant”. These cut-offs in the pre-registered study were based on recently published recommendations^75^. All calculations were performed using Stata 15. Linear regressions were estimated using robust standard errors, as implemented in Stata.

## Supplementary information

Supplementary material

## Data Availability

The data that support the findings of this study are available from the German Socio-Economic Panel Innovation Sample (SOEP-IS) team, but restrictions apply to the availability of the data, which were used under license for the current study, and so are not publicly available. Data are however available from the corresponding author upon reasonable request and with permission of the German Socio-Economic Panel Innovation Sample (SOEP-IS) team.
