# Supplementary material for: Depression, neuroticism and 2D:4D ratio: evidence from a large, representative sample
Source: Sci Rep. 2020 Jul 7;10:11136. doi: 10.1038/s41598-020-67882-x (PMC7341800; doi:10.1038/s41598-020-67882-x)
Supplement: Supplementary file 1 — Supplementary material [file 41598_2020_67882_MOESM1_ESM.pdf]

Depression, Neuroticism and 2D:4D Ratio: Evidence from a Large, Representative Sample (Supplementary Materials)

Leopold Lautenbacher & Levent Neyse (2020)

1 Previous findings on 2D:4D and depression or neuroticism

We were able to identify 16 previous papers that analyzed 2D:4D in the context of depression or neuroticism. The results are summarized in Supplementary Table S1 which shows the direction of the various associations and which associations were considered statistically significant based on a cut-off of $p < 0.05$. For the sake of space, only the reference numbers are used to refer to each publication included in the table. These reference numbers refer to the reference section of the supplementary materials which can be found at the end of this document. They should not be mistaken for the reference numbers used in the main article.

Supplementary Table S1. Associations of 2D:4D with depression or neuroticism in previous studies.

Reference	n	Right hand 2D:4D		Left hand 2D:4D		Mean 2D:4D	
		Males	Females	Males	Females	Males	Females
Depression							
[1]	335	?	?				
[2]^a	165	$-$	$+$				
[3]	298	$+^*$	$+$				
[4]	138					$-$	$+$
[6]	31		$+^*$		$+^*$		
[12]	102					$-$	$-$
[13]	137	$-^*$	$-$	$+$	$+$		
[15]	274	$+$	$-$	$+$	$-$		
[17]	128	?	$-^*$				
[18]	594	$-^*$	$+$				
[20]	245	$-$		$-$			
[21]	196	$+^*$	$-$	$+$	$+$		
Neuroticism							
[2]^a	165	$-$	$+$				
[2]^b	100	$+$	$+$	$+$	$+$		
[5]	80	$+$	$+^*$	$+$	$+$		
[7]	1991					?	?
[11]	23	$+^*$	$+$	$+^*$	$+$		
[16]	448	$-$	$+^*$	$-$	$+^*$		

Note: $+$ = positive association, i.e., a more "feminine" digit ratio was associated with higher depression / neuroticism rates, the direction hypothesized in the present study; $-$ = negative association, opposite to the direction hypothesized; ? = the effect direction is not known to the authors of the present study, but it is clear that the association was tested due to statements about statistical significance. For empty cells, no corresponding results could be found. An asterisk (*) indicates that the effect was considered statistically significant ($p < 0.05$). All associations presented without asterisks were nonsignificant ($p > 0.05$). Austin and colleagues [2] reported separate results for two samples which are differentiated as [2]^a and [2]^b. The 23 observations listed as the sample size for Manning & Fink [11] represent aggregated mean data for 23 nations. The results of Li et al. [6] are restricted to cynomolgus monkeys.

2 Additional analyses: differences depending on consent to 2D:4D measurement

We used data from the Innovation Sample of the German Socio-Economic Panel Study up to the survey year 2018 [14]. In 2018, 3,509 of 3,958 individuals gave their consent to having their digit ratios measured. On the variables we studied, there were no significant differences between participants who consented to getting their digit ratios measured and those who declined consent. The corresponding tests are reported in Supplementary Table S2. For biological sex and history of depression, chi-squared (χ^2) is reported. For neuroticism values and scores on the Patient Health Questionnaire-2 (PHQ-2) [9], t-values are presented with p-values for two-tailed tests.

Supplementary Table S2. Tests of differences depending on consent to 2D:4D measurement.

Variable	n	t / χ^2	p
Biological sex	3,918	2.01	.157
HIST	3,918	<0.001	.996
NEUR	3,822	0.45	.651
PHQ-2	1,399	0.02	.987

Note: HIST = history of depression, NEUR = neuroticism, measured using the Big Five Inventory-SOEP, PHQ-2 = Patient Health Questionnaire-2, a measure of acute depressive symptoms. All tests compared those participants who consented to getting their digit ratios measured with those who declined consent.

3 Additional analyses: controlling for age

Due to observing small but consistent negative pairwise correlations of age with all of the 2D:4D measures and with two of the outcome measures, exploratory analyses were conducted which included age as an additional predictor. The sample of 3,389 individuals had an age range between 18 and 98 years ($M = 55, SD = 18$). For the regression analyses, the age variable was standardized as well to improve comparability of effect sizes. The results of these additional exploratory analyses for right hand 2D:4D, left hand 2D:4D and the mean 2D:4D of both hands are shown in Supplementary Table S3, Supplementary Table S4 and Supplementary Table S5, respectively. Most notably, no association with any 2D:4D measure had a *p*-value below our threshold for suggestive evidence when controlling for age.

As several previous studies analyzed university students, we also repeated the exploratory analyses with an interaction term on a subsample restricted to participants under the age of 30 to account for a possible age-specific effect in younger people. The results of these additional analyses for right hand 2D:4D, left hand 2D:4D and the mean 2D:4D of both hands are shown in Supplementary Table S6, Supplementary Table S7 and Supplementary Table S8, respectively. There was no suggestive or significant evidence for any association with 2D:4D in this age-restricted subsample. However, sample sizes were severely reduced. This likely affected the accuracy of the estimates and the statistical power. The confidence intervals were noticeably wider compared with those estimated using the full sample. Interestingly, the effect directions of 2D:4D reversed for the depression measures, indicating negative associations, but considering the inaccuracy involved, the relevance of this observation is debatable.

Supplementary Table S3. Additional analyses controlling for age, using right hand 2D:4D data.

Variable	HIST ($n = 3,389$)		NEUR ($n = 3,316$)		PHQ-2 ($n = 1,210$)	
	OR [CI]	*p*	β [CI]	*p*	β [CI]	*p*
R2D:4D	1.08 [0.95, 1.23]	0.24	0.02 [-0.01, 0.06]	0.17	0.04 [-0.01, 0.10]	0.11
FEM	1.98 [1.49, 2.62]	<0.001	0.49 [0.42, 0.56]	<0.001	-0.04 [-0.15, 0.07]	0.48
AGE	0.93 [0.81, 1.06]	0.25	-0.09 [-0.12, -0.06]	<0.001	-0.15 [-0.21, -0.09]	<0.001
R2D:4D	1.05 [0.83, 1.33]	0.68	0.02 [-0.03, 0.07]	0.38	0.04 [-0.04, 0.12]	0.34
FEM	1.97 [1.49, 2.62]	<0.001	0.49 [0.42, 0.56]	<0.001	-0.04 [-0.15, 0.07]	0.48
R2D:4D x FEM	1.04 [0.78, 1.38]	0.78	<-0.01 [-0.07, 0.07]	0.95	0.01 [-0.10, 0.12]	0.90
AGE	0.93 [0.81, 1.06]	0.25	-0.09 [-0.12, -0.06]	<0.001	-0.15 [-0.21, -0.09]	<0.001

Note: CI = 95% confidence interval, FEM = female sex, HIST = history of depression, NEUR = neuroticism, *OR* = odds ratio, PHQ-2 = Patient Health Questionnaire-2, R2D:4D = the length of the second digit divided by the length of the fourth digit of the right hand. β indicates the change in the *z*-standardized outcome variables for a change of one standard deviation in 2D:4D (or age) while controlling for biological sex and age (or 2D:4D) or the change in the case of female instead of male participants while controlling for age and 2D:4D.

Supplementary Table S4. Additional analyses controlling for age, using left hand 2D:4D data.

Variable	HIST ($n = 3,387$)		NEUR ($n = 3,313$)		PHQ-2 ($n = 1,204$)	
	OR [CI]	*p*	β [CI]	*p*	β [CI]	*p*
L2D:4D	1.13 [0.99, 1.29]	0.08	0.03 [<-0.01, 0.06]	0.09	<0.01 [-0.05, 0.06]	0.87
FEM	1.98 [1.49, 2.62]	<0.001	0.49 [0.42, 0.56]	<0.001	-0.03 [-0.14, 0.08]	0.60
AGE	0.92 [0.81, 1.05]	0.22	-0.09 [-0.13, -0.06]	<0.001	-0.16 [-0.22, -0.09]	<0.001
L2D:4D	1.07 [0.85, 1.36]	0.56	0.04 [-0.01, 0.09]	0.09	<-0.01 [-0.10, 0.09]	0.92
FEM	1.97 [1.48, 2.62]	<0.001	0.49 [0.42, 0.56]	<0.001	-0.03 [-0.14, 0.08]	0.60
L2D:4D x FEM	1.07 [0.81, 1.43]	0.62	-0.02 [-0.09, 0.04]	0.46	0.02 [-0.10, 0.13]	0.78
AGE	0.93 [0.81, 1.05]	0.22	-0.09 [-0.13, -0.06]	<0.001	-0.16 [-0.22, -0.09]	<0.001

Note: CI = 95% confidence interval, FEM = female sex, HIST = history of depression, L2D:4D = the length of the second digit divided by the length of the fourth digit of the left hand, NEUR = neuroticism, *OR* = odds ratio, PHQ-2 = Patient Health Questionnaire-2. β indicates the change in the *z*-standardized outcome variables for a change of one standard deviation in 2D:4D (or age) while controlling for biological sex and age (or 2D:4D) or the change in the case of female instead of male participants while controlling for age and 2D:4D.

Supplementary Table S5. Additional analyses controlling for age, using the mean 2D:4D ratio of both hands.

Variable	HIST ($n = 3,337$)		NEUR ($n = 3,264$)		PHQ-2 ($n = 1,185$)	
	OR [CI]	*p*	β [CI]	*p*	β [CI]	*p*
M2D:4D	1.13 [0.99, 1.30]	0.07	0.03 [<-0.01, 0.07]	0.053	0.03 [-0.03, 0.08]	0.33
FEM	1.95 [1.46, 2.59]	<0.001	0.49 [0.42, 0.55]	<0.001	-0.04 [-0.16, 0.07]	0.45
AGE	0.92 [0.80, 1.05]	0.22	-0.09 [-0.12, -0.06]	<0.001	-0.16 [-0.22, -0.09]	<0.001
M2D:4D	1.07 [0.85, 1.37]	0.56	0.04 [-0.01, 0.09]	0.08	0.02 [-0.07, 0.10]	0.70
FEM	1.94 [1.46, 2.58]	<0.001	0.49 [0.42, 0.55]	<0.001	-0.04 [-0.16, 0.07]	0.46
M2D:4D x FEM	1.08 [0.81, 1.44]	0.60	-0.02 [-0.09, 0.05]	0.61	0.02 [-0.09, 0.13]	0.74
AGE	0.92 [0.80, 1.05]	0.22	-0.09 [-0.12, -0.06]	<0.001	-0.16 [-0.22, -0.09]	<0.001

Note: CI = 95% confidence interval, FEM = female sex, HIST = history of depression, M2D:4D = the mean of the digit ratios of the right hand and left hand, NEUR = neuroticism, *OR* = odds ratio, PHQ-2 = Patient Health Questionnaire-2. β indicates the change in the *z*-standardized outcome variables for a change of one standard deviation in 2D:4D (or age) while controlling for biological sex and age (or 2D:4D) or the change in the case of female instead of male participants while controlling for age and 2D:4D.

Supplementary Table S6. Additional analyses restricted to participants under the age of 30, using right hand 2D:4D data.

Variable	HIST ($n = 389$)		NEUR ($n = 340$)		PHQ-2 ($n = 63$)	
	OR [CI]	*p*	β [CI]	*p*	β [CI]	*p*
R2D:4D	0.70 [0.33, 1.52]	0.37	0.04 [-0.13, 0.20]	0.66	-0.04 [-0.38, 0.31]	0.84
FEM	1.82 [0.73, 4.54]	0.20	0.47 [0.28, 0.67]	<0.001	0.06 [-0.42, 0.53]	0.81
R2D:4D x FEM	1.26 [0.48, 3.27]	0.64	0.11 [-0.11, 0.32]	0.33	0.16 [-0.30, 0.62]	0.50

Note: CI = 95% confidence interval, FEM = female sex, HIST = history of depression, NEUR = neuroticism, *OR* = odds ratio, PHQ-2 = Patient Health Questionnaire-2, R2D:4D = the length of the second digit divided by the length of the fourth digit of the right hand. β indicates the change in the z-standardized outcome variables for a change of one standard deviation in 2D:4D while controlling for biological sex or the change in the case of female instead of male participants while controlling for 2D:4D.

Supplementary Table S7. Additional analyses restricted to participants under the age of 30, using left hand 2D:4D data.

Variable	HIST ($n = 386$)		NEUR ($n = 336$)		PHQ-2 ($n = 63$)	
	OR [CI]	*p*	β [CI]	*p*	β [CI]	*p*
L2D:4D	0.75 [0.34, 1.66]	0.47	0.03 [-0.12, 0.18]	0.65	-0.04 [-0.48, 0.41]	0.87
FEM	1.79 [0.72, 4.46]	0.21	0.48 [0.28, 0.67]	<0.001	0.04 [-0.40, 0.49]	0.85
L2D:4D x FEM	1.48 [0.56, 3.95]	0.43	0.07 [-0.14, 0.27]	0.53	0.16 [-0.43, 0.75]	0.59

Note: CI = 95% confidence interval, FEM = female sex, HIST = history of depression, L2D:4D = the length of the second digit divided by the length of the fourth digit of the left hand, NEUR = neuroticism, *OR* = odds ratio, PHQ-2 = Patient Health Questionnaire-2. β indicates the change in the z-standardized outcome variables for a change of one standard deviation in 2D:4D while controlling for biological sex or the change in the case of female instead of male participants while controlling for 2D:4D.

Supplementary Table S8. Additional analyses restricted to participants under the age of 30, using mean 2D:4D data.

Variable	HIST ($n = 385$)		NEUR ($n = 336$)		PHQ-2 ($n = 63$)	
	OR [CI]	*p*	β [CI]	*p*	β [CI]	*p*
M2D:4D	0.69 [0.33, 1.46]	0.33	0.04 [-0.11, 0.20]	0.60	-0.04 [-0.41, 0.34]	0.85
FEM	1.85 [0.74, 4.65]	0.19	0.47 [0.27, 0.67]	<0.001	0.05 [-0.41, 0.50]	0.83
M2D:4D x FEM	1.46 [0.57, 3.75]	0.43	0.10 [-0.11, 0.31]	0.34	0.15 [-0.32, 0.63]	0.52

Note: CI = 95% confidence interval, FEM = female sex, HIST = history of depression, M2D:4D = the mean of the digit ratios of the right hand and left hand, NEUR = neuroticism, *OR* = odds ratio, PHQ-2 = Patient Health Questionnaire-2. β indicates the change in the z-standardized outcome variables for a change of one standard deviation in 2D:4D while controlling for biological sex or the change in the case of female instead of male participants while controlling for 2D:4D.

4 Additional analyses: including quadratic terms

Further analyses using quadratic terms yielded similarly small effect sizes as the analyses reported in the main article. The results of these additional analyses with quadratic terms for right hand 2D:4D and left hand 2D:4D are shown in Supplementary Table S9 and Supplementary Table S10, respectively. The results for the quadratic term of right hand 2D:4D reached our threshold for suggestive evidence (history of depression: $p = 0.03$; neuroticism: $p = 0.04$; PHQ-2: $p = 0.048$). The results are visualized in the following section ("Illustrative figures") of these supplementary materials.

Supplementary Table S9. Additional analyses using the right hand 2D:4D with a quadratic term.

Variable	HIST ($n = 3,389$)		NEUR ($n = 3,316$)		PHQ-2 ($n = 1,210$)	
	OR [CI]	*p*	β [CI]	*p*	β [CI]	*p*
R2D:4D	1.11 [0.95, 1.28]	0.18	0.03 [-0.01, 0.06]	0.12	0.04 [-0.01, 0.10]	0.13
FEM	1.99 [1.50, 2.63]	<0.001	0.49 [0.43, 0.56]	<0.001	-0.03 [-0.15, 0.08]	0.58
R2D:4D^2	0.90 [0.81, 0.99]	0.03	-0.02 [-0.04, <-0.01]	0.04	-0.03 [-0.06, <-0.01]	0.048

Note: CI = 95% confidence interval, FEM = female sex, HIST = history of depression, NEUR = neuroticism, PHQ-2 = Patient Health Questionnaire-2, *OR* = odds ratio, R2D:4D = the length of the second digit divided by the length of the fourth digit of the right hand. β indicates the change in the z-standardized outcome variables for a change of one standard deviation in 2D:4D while controlling for biological sex or the change in the case of female instead of male participants while controlling for 2D:4D.

Supplementary Table S10. Additional analyses using the left hand 2D:4D with a quadratic term.

Variable	HIST ($n = 3,387$)		NEUR ($n = 3,313$)		PHQ-2 ($n = 1,204$)	
	OR [CI]	*p*	β [CI]	*p*	β [CI]	*p*
L2D:4D	1.12 [0.99, 1.28]	0.08	0.03 [<-0.01, 0.07]	0.051	0.01 [-0.05, 0.07]	0.77
FEM	1.97 [1.49, 2.62]	<0.001	0.49 [0.42, 0.56]	<0.001	-0.02 [-0.14, 0.09]	0.72
L2D:4D^2	1.02 [0.94, 1.10]	0.65	<0.01 [-0.02, 0.02]	0.79	-0.01 [-0.04, 0.02]	0.59

Note: CI = 95% confidence interval, FEM = female sex, HIST = history of depression, L2D:4D = the length of the second digit divided by the length of the fourth digit of the left hand, NEUR = neuroticism, PHQ-2 = Patient Health Questionnaire-2, *OR* = odds ratio. β indicates the change in the z-standardized outcome variables for a change of one standard deviation in 2D:4D while controlling for biological sex or the change in the case of female instead of male participants while controlling for 2D:4D.

5 Illustrative figures

Supplementary Figure S1 illustrates the null result for the interaction effect in the exploratory logistic regression model. As the exploratory logistic regression results were very similar across 2D:4D measures, the figure only illustrates the right hand 2D:4D data for the sake of space. The results for the regression models with quadratic terms (R2D:4D^2 and L2D:4D^2) are visualized in Supplementary Fig. S2 and Supplementary Fig. S3. There was suggestive evidence for quadratic associations with the right hand 2D:4D data, but it is barely visible, illustrating the very small effect sizes involved.

Supplementary Figure S1. Marginal effects in the exploratory logistic regression, using right hand 2D:4D.

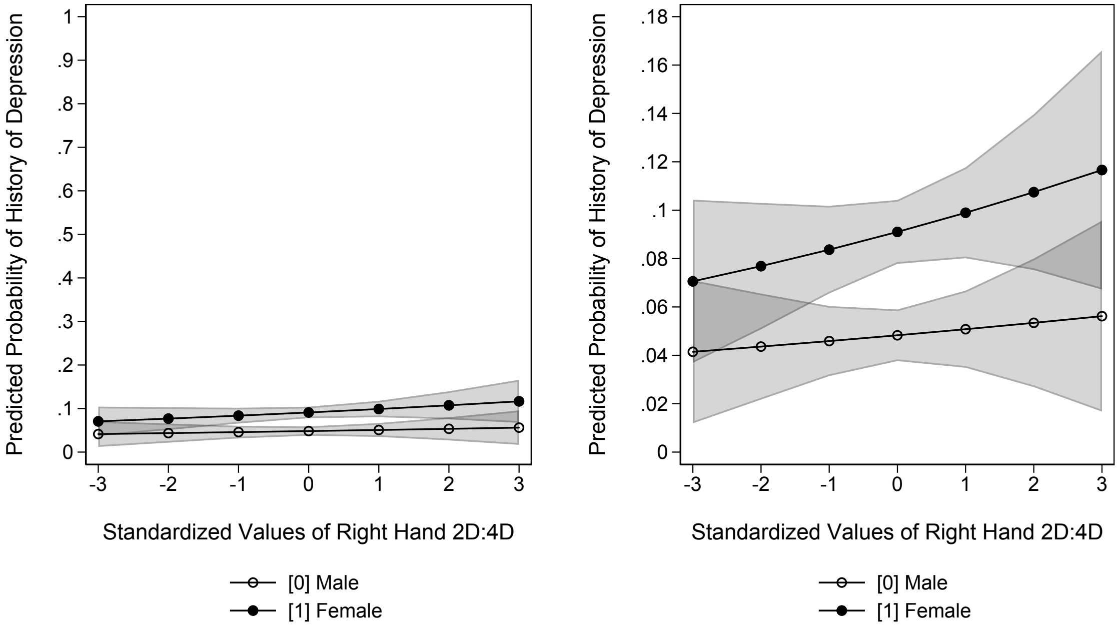

Note: 2D:4D = the length of the second digit divided by the length of the fourth digit of the hand. In the left graph, the results are shown in the context of the full range of probability. In the right graph, the y-axis was capped at .18 for illustrative purposes. The area around a line illustrates the 95% confidence interval for the corresponding estimate. Differences in the slopes would indicate an interaction effect of 2D:4D with sex. The range of standardized 2D:4D values displayed includes 99% of the sample used for the regression.

Supplementary Figure S2. Marginal effects in the regression models with a quadratic term for the right hand (R2D:4D^2).

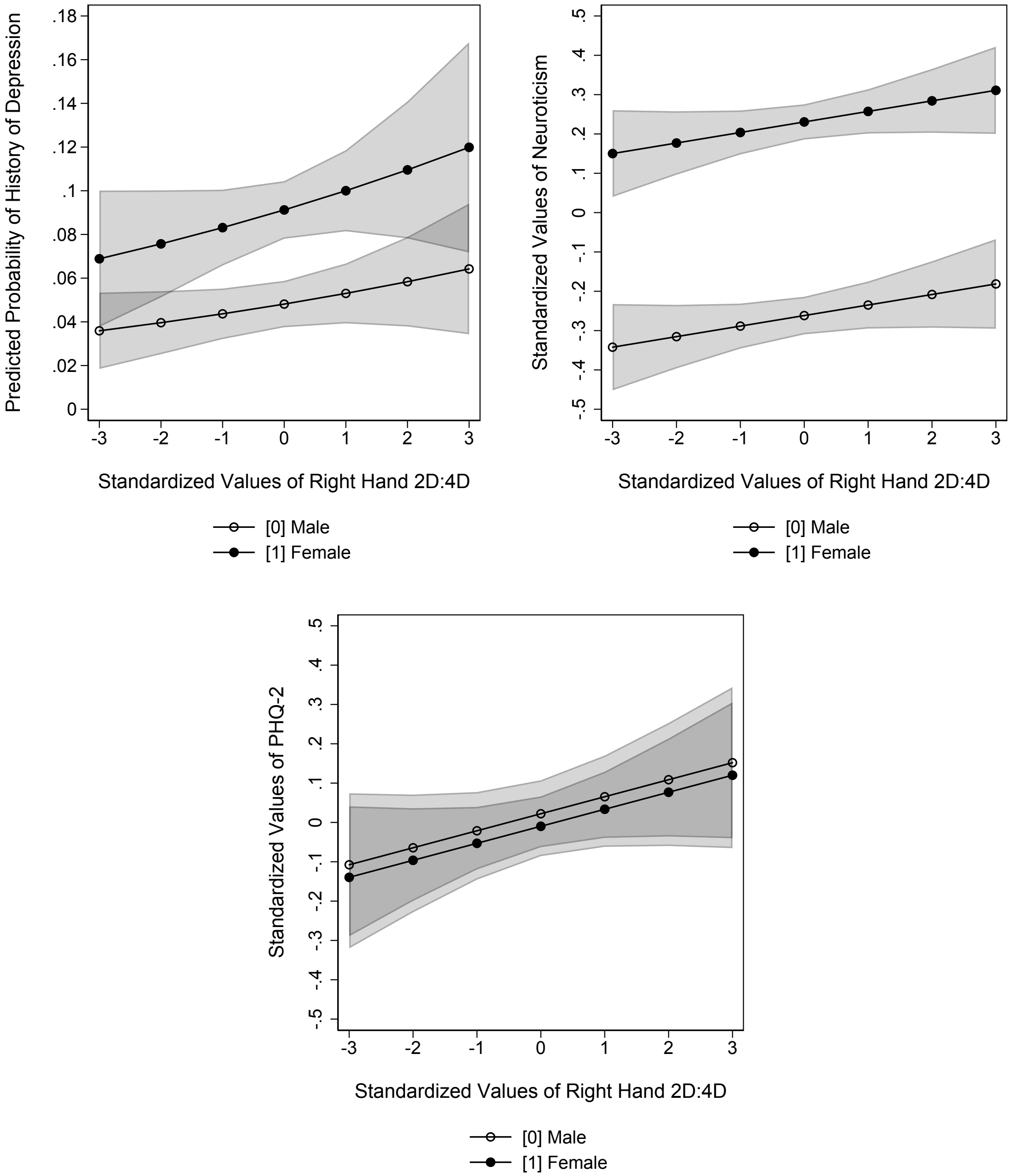

Note: 2D:4D = the length of the second digit divided by the length of the fourth digit of the hand. The y-axis was capped in each graph for illustrative purposes. The area around a line illustrates the 95% confidence interval for the corresponding estimate. The range of standardized 2D:4D values displayed includes 99% of the sample used for the regression models.

Supplementary Figure S3. Marginal effects in the regression models with a quadratic term for the left hand (L2D:4D^2).

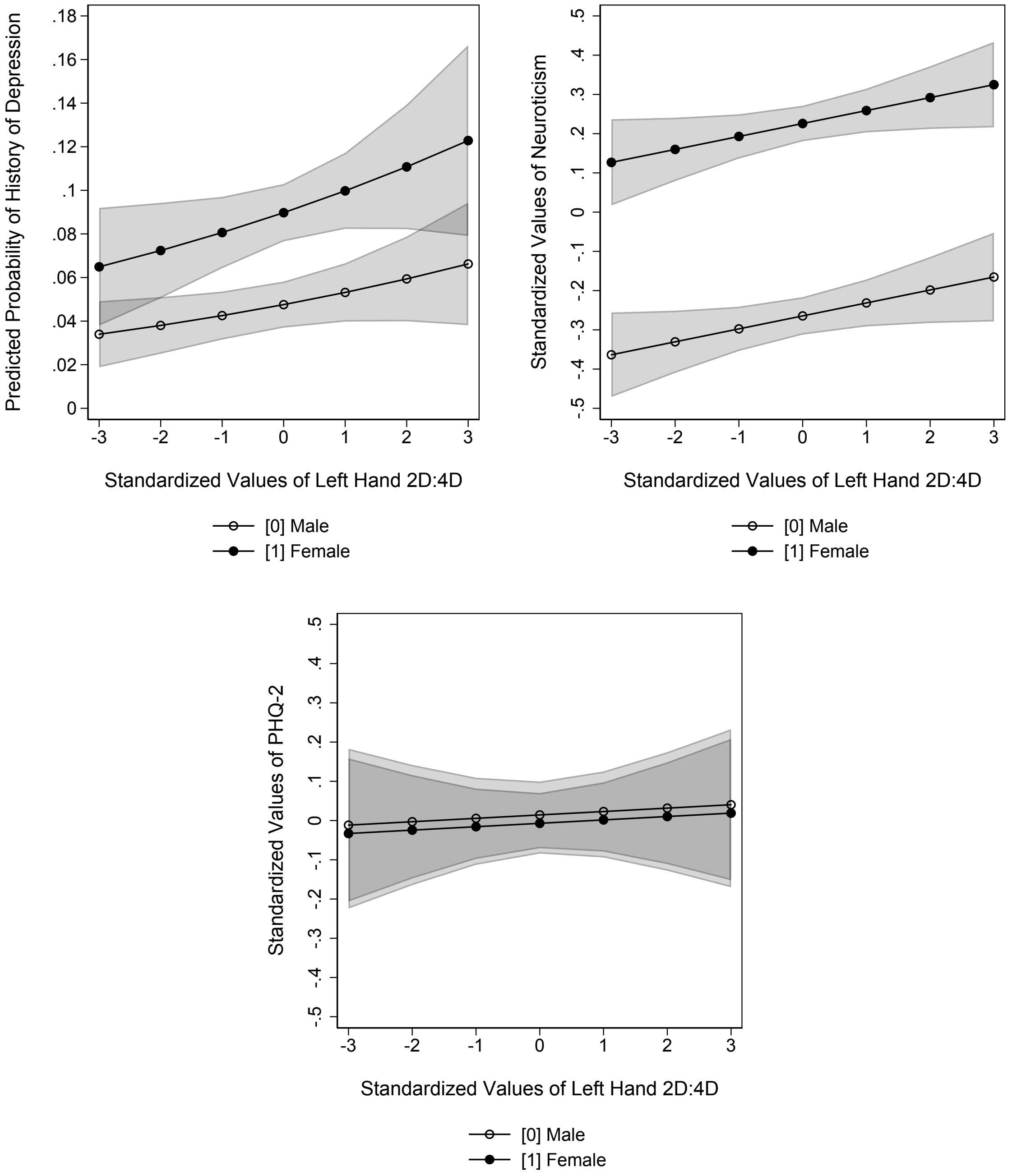

Note: 2D:4D = the length of the second digit divided by the length of the fourth digit of the hand. The y-axis was capped in each graph for illustrative purposes. The area around a line illustrates the 95% confidence interval for the corresponding estimate. The range of standardized 2D:4D values displayed includes 99% of the sample used for the regression models.

6 Additional analyses: multilevel modeling accounting for interviewer effects

To investigate the influence of systematic differences between interviewers, we also repeated the exploratory analyses with an interaction term using multilevel modeling, grouping 2D:4D observations by interviewers. Multilevel modeling was performed using the *meglm* command in Stata, estimating normally distributed random effects. Robust standard errors were estimated for neuroticism and PHQ-2 scores. The family-link combination of "bernoulli" and "logit" was specified for history of depression. Due to exclusions of participants, not all of the 263 interviewers who measured 2D:4D ratios in 2018 are included. The number of interviewers ranged from 168 to 239 depending on the combination of 2D:4D measure and outcome variable. The number of observations per interviewer ranged from one to 61, with an average number of observations per interviewer of 14 for history of depression and neuroticism and seven for the PHQ-2.

The main results of these additional exploratory analyses using multilevel modeling for right hand 2D:4D, left hand 2D:4D and the mean 2D:4D of both hands are shown in Supplementary Table S11, Supplementary Table S12 and Supplementary Table S13, respectively. Notably, when grouping 2D:4D observations by interviewers, the associations with neuroticism which had p-values below 0.05 in the analyses reported in the main article do not reach this threshold for suggestive evidence anymore ($p > 0.05$ for all associations with 2D:4D measures).

Supplementary Table S11. Additional analyses grouping right hand 2D:4D observations by interviewers.

Variable	HIST ($n_1 = 3,389$; $n_2 = 238$)		NEUR ($n_1 = 3,316$; $n_2 = 238$)		PHQ-2 ($n_1 = 1,210$; $n_2 = 170$)	
	OR [CI]	*p*	β [CI]	*p*	β [CI]	*p*
R2D:4D	1.05 [0.83, 1.35]	0.65	0.02 [-0.02, 0.07]	0.32	0.05 [-0.04, 0.13]	0.30
FEM	2.00 [1.50, 2.66]	<0.001	0.49 [0.42, 0.57]	<0.001	-0.02 [-0.12, 0.08]	0.68
R2D:4D x FEM	1.04 [0.78, 1.39]	0.79	<-0.01 [-0.06, 0.06]	0.91	0.03 [-0.07, 0.13]	0.56

Note: CI = 95% confidence interval, FEM = female sex, HIST = history of depression, n_1 = number of individual participants, n_2 = number of groups based on interviewers, NEUR = neuroticism, *OR* = odds ratio, PHQ-2 = Patient Health Questionnaire-2, R2D:4D = the length of the second digit divided by the length of the fourth digit of the right hand. β indicates the change in the z-standardized outcome variables for a change of one standard deviation in 2D:4D while controlling for biological sex or the change in the case of female instead of male participants while controlling for 2D:4D.

Supplementary Table S12. Additional analyses grouping left hand 2D:4D observations by interviewers.

Variable	HIST ($n_1 = 3,387$; $n_2 = 239$)		NEUR ($n_1 = 3,313$; $n_2 = 239$)		PHQ-2 ($n_1 = 1,204$; $n_2 = 172$)	
	OR [CI]	*p*	β [CI]	*p*	β [CI]	*p*
L2D:4D	1.07 [0.84, 1.36]	0.57	0.05 [-0.01, 0.10]	0.09	-0.01 [-0.09, 0.07]	0.83
FEM	1.98 [1.49, 2.65]	<0.001	0.49 [0.42, 0.57]	<0.001	-0.02 [-0.12, 0.09]	0.76
L2D:4D x FEM	1.09 [0.82, 1.45]	0.56	-0.02 [-0.09, 0.05]	0.57	0.06 [-0.04, 0.16]	0.27

Note: CI = 95% confidence interval, FEM = female sex, HIST = history of depression, L2D:4D = the length of the second digit divided by the length of the fourth digit of the left hand, n_1 = number of individual participants, n_2 = number of groups based on interviewers, NEUR = neuroticism, *OR* = odds ratio, PHQ-2 = Patient Health Questionnaire-2. β indicates the change in the z-standardized outcome variables for a change of one standard deviation in 2D:4D while controlling for biological sex or the change in the case of female instead of male participants while controlling for 2D:4D.

Supplementary Table S13. Additional analyses grouping mean 2D:4D observations by interviewers.

Variable	HIST ($n_1 = 3,337$; $n_2 = 237$)		NEUR ($n_1 = 3,264$; $n_2 = 237$)		PHQ-2 ($n_1 = 1,185$; $n_2 = 168$)	
	OR [CI]	*p*	β [CI]	*p*	β [CI]	*p*
M2D:4D	1.08 [0.85, 1.38]	0.54	0.05 [<-0.01, 0.10]	0.07	0.02 [-0.06, 0.09]	0.70
FEM	1.96 [1.47, 2.62]	<0.001	0.49 [0.41, 0.57]	<0.001	-0.03 [-0.13, 0.08]	0.61
M2D:4D x FEM	1.09 [0.81, 1.46]	0.57	-0.02 [-0.09, 0.05]	0.61	0.06 [-0.04, 0.16]	0.26

Note: CI = 95% confidence interval, FEM = female sex, HIST = history of depression, M2D:4D = the mean of the digit ratios of the right hand and left hand, n_1 = number of individual participants, n_2 = number of groups based on interviewers, NEUR = neuroticism, *OR* = odds ratio, PHQ-2 = Patient Health Questionnaire-2. β indicates the change in the z-standardized outcome variables for a change of one standard deviation in 2D:4D while controlling for biological sex or the change in the case of female instead of male participants while controlling for 2D:4D.

7 Additional analyses: digit asymmetry measures

To inspect our 2D:4D data for anomalies, digit asymmetry measures were analyzed. For this, we focused on replicating three previous observations reported in the literature: a sex difference in left- and rightward asymmetry [19], a sex difference in composite asymmetry [10] and a quadratic relationship between 2D:4D and composite asymmetry [10].

Similar to the methodology of Voracek, Offenmüller & Dressler [19], participants were grouped in three categories depending on their signed asymmetry scores between right hand and left hand for the second and fourth digit: participants with scores above 0.25mm for both digits were categorized as having rightward asymmetry, participants with scores below -0.25mm for both digits were categorized as having leftward asymmetry. The remainder was categorized as an "all other" group. Using a chi-squared (χ^2) test, we analyzed whether there was a statistical dependency between biological sex and directional asymmetry. Our results replicate the finding of Voracek and colleagues [19]: there was a significant relationship ($p < 0.005$), with leftward asymmetry being more frequent in men and rightward asymmetry being more frequent in women. The 3x2 data used for the analysis is shown in Supplementary Table S14.

Similar to the methodology of Manning & Fink [10], we computed the relative asymmetries for the second and fourth digit, then summed these and divided by two to obtain a composite asymmetry score ($Asy_{2,4}$). Then, as done in the previous study, we restricted the range of second digit and fourth digit asymmetry from perfect symmetry (0) to 10% asymmetry to remove extreme cases. Our analysis using a two-tailed t-test did not replicate the finding of a sex difference in composite asymmetry ($d = -0.03$, $p = 0.45$). The average composite asymmetry was approximately 3% in both female and male participants, compared to 2% in female and 1.8% in male participants of the previous study by Manning & Fink [10]. However, standard deviations for composite asymmetry were very similar compared to previously reported estimates [10]. Furthermore, the finding of a positive quadratic relationship between 2D:4D and composite asymmetry [10] was replicated in both right hand and left hand data ($p < 0.005$), using polynomial regression models. The results of these analyses on composite asymmetry are presented in Supplementary Table S15 and Supplementary Table S16.

The analyses of digit asymmetry measures did not indicate any major anomalies in our 2D:4D data compared to previous datasets, further supporting the notion that reasonably accurate 2D:4D measurements were collected.

Supplementary Table S14. Test of a sex difference in left- and rightward asymmetry using a chi-squared test.

	Leftward asymmetry	All other	Rightward asymmetry	Total	χ^2	p
					11.74	0.003
Female	334	815	406	1,555		
Male	309	1,017	508	1,834		
Total	643	1,832	914	3,389		

Note: Participants with directional asymmetry scores above 0.25mm for both digits were categorized as having rightward asymmetry, participants with scores below -0.25mm for both digits were categorized as having leftward asymmetry. The remainder was categorized as an "all other" group.

Supplementary Table S15. Test of a sex difference in composite asymmetry using a two-tailed t-test.

Variable	*n*	*M*	*SD*	*t*	*d*	*p*
$Asy_{2,4}$				-0.75	-0.03	0.45
Female	1,519	0.02964	0.0166			
Male	1,278	0.02917	0.0169			

Note: $Asy_{2,4}$ = composite asymmetry, M = mean, SD = standard deviation.

Supplementary Table S16. Additional analyses of composite asymmetry using polynomial regression models.

Variable	$Asy_{2,4}$ in females ($n = 1,519$)		$Asy_{2,4}$ in males ($n = 1,278$)	
	β [CI]	*p*	β [CI]	*p*
R2D:4D	-0.0003 [-0.0013, 0.0006]	0.47	-0.0004 [-0.0015, 0.0007]	0.49
R2D:4D^2	0.0018 [0.0011, 0.0024]	<0.001	0.0017 [0.0009, 0.0025]	<0.001
L2D:4D	-0.0006 [-0.0016, 0.0003]	0.20	<-0.0001 [-0.0011, 0.0010]	0.95
L2D:4D^2	0.0018 [0.0011, 0.0025]	<0.001	0.0025 [0.0017, 0.0033]	<0.001

Note: 2D:4D = the length of the second digit divided by the length of the fourth digit of the hand (R = right hand, L = left hand), $Asy_{2,4}$ = composite asymmetry, CI = 95% confidence interval. β indicates the change in composite asymmetry for a change of one standard deviation in 2D:4D, e.g., a β of 0.01 would indicate a change of one standard deviation in 2D:4D being associated with a 1% increase in asymmetry.

8 Additional analyses: interaction between 2D:4D and history of depression

Based on the theorizing that higher prenatal testosterone exposure, associated with increased activation to positive stimuli [8], may be a protective factor in brain development, associations of 2D:4D with behavior within the clinical population could possibly differ from associations in the rest of the population. To investigate this, additional analyses including history of depression and an interaction term between 2D:4D and history of depression as predictors were performed for the neuroticism and PHQ-2 outcome variables. The results of these additional analyses including an interaction term with history of depression for right hand 2D:4D, left hand 2D:4D and the mean 2D:4D of both hands are shown in Supplementary Table S17, Supplementary Table S18 and Supplementary Table S19, respectively.

There was suggestive evidence for one interaction effect, but it was restricted to the right hand data and the neuroticism outcome, with a p-value barely below the threshold ($p = 0.046$). The p-values for the left hand and mean 2D:4D interactions were considerably larger. Therefore, it seems plausible that this suggestive evidence for a single interaction effect in the right hand data could represent a false positive. Due to these limitations and the limited usefulness for informing the evaluation of our hypotheses, these results are not further discussed in the main article. It should be noted that the main effects on neuroticism in the right hand and left hand data were surprisingly similar, resulting in identical rounded values.

Supplementary Table S17. Additional analyses with interaction between right hand 2D:4D and history of depression.

Variable	NEUR ($n = 3,316$)		PHQ-2 ($n = 1,210$)	
	β [CI]	p	β [CI]	p
R2D:4D	0.03 [<-0.01, 0.07]	0.07	0.02 [-0.03, 0.07]	0.43
FEM	0.46 [0.40, 0.53]	<0.001	-0.08 [-0.19, 0.03]	0.17
HIST	0.71 [0.59, 0.83]	<0.001	1.01 [0.73, 1.29]	<0.001
R2D:4D x HIST	-0.14 [-0.28, <-0.01]	0.046	0.29 [-0.05, 0.63]	0.10

Note: R2D:4D = the length of the second digit divided by the length of the fourth digit of the right hand, CI = 95% confidence interval, FEM = female sex, HIST = history of depression, NEUR = neuroticism, *OR* = odds ratio, PHQ-2 = Patient Health Questionnaire-2. β indicates the change in the z-standardized outcome variables for a change of one standard deviation in 2D:4D while controlling for biological sex and history of depression, the change in the case of female instead of male participants while controlling for 2D:4D and history of depression or the change in the case of cases instead of controls while controlling for 2D:4D and biological sex.

Supplementary Table S18. Additional analyses with interaction between left hand 2D:4D and history of depression.

Variable	NEUR ($n = 3,313$)		PHQ-2 ($n = 1,204$)	
	β [CI]	p	β [CI]	p
L2D:4D	0.03 [<-0.01, 0.07]	0.07	<-0.01 [-0.05, 0.05]	0.95
FEM	0.46 [0.40, 0.53]	<0.001	-0.07 [-0.18, 0.04]	0.20
HIST	0.71 [0.58, 0.83]	<0.001	1.06 [0.75, 1.36]	<0.001
L2D:4D x HIST	-0.06 [-0.18, 0.07]	0.37	0.04 [-0.28, 0.36]	0.79

Note: L2D:4D = the length of the second digit divided by the length of the fourth digit of the left hand, CI = 95% confidence interval, FEM = female sex, HIST = history of depression, NEUR = neuroticism, *OR* = odds ratio, PHQ-2 = Patient Health Questionnaire-2. β indicates the change in the z-standardized outcome variables for a change of one standard deviation in 2D:4D while controlling for biological sex and history of depression, the change in the case of female instead of male participants while controlling for 2D:4D and history of depression or the change in the case of cases instead of controls while controlling for 2D:4D and biological sex.

Supplementary Table S19. Additional analyses with interaction between mean 2D:4D and history of depression.

Variable	NEUR ($n = 3,264$)		PHQ-2 ($n = 1,185$)	
	β [CI]	p	β [CI]	p
M2D:4D	0.04 [0.01, 0.07]	0.02	0.01 [-0.04, 0.06]	0.74
FEM	0.46 [0.39, 0.52]	<0.001	-0.08 [-0.19, 0.03]	0.17
HIST	0.71 [0.58, 0.83]	<0.001	1.00 [0.70, 1.30]	<0.001
M2D:4D x HIST	-0.12 [-0.26, 0.02]	0.10	0.22 [-0.16, 0.60]	0.26

Note: M2D:4D = the mean of the digit ratios of the right hand and left hand, CI = 95% confidence interval, FEM = female sex, HIST = history of depression, NEUR = neuroticism, *OR* = odds ratio, PHQ-2 = Patient Health Questionnaire-2. β indicates the change in the z-standardized outcome variables for a change of one standard deviation in 2D:4D while controlling for biological sex and history of depression, the change in the case of female instead of male participants while controlling for 2D:4D and history of depression or the change in the case of cases instead of controls while controlling for 2D:4D and biological sex.

References

1. Au Yeung, L. & Tse, W. S. Why does digit ratio research fail to give any implication regarding the organizational effect of prenatal androgen? *Journal of Individual Differences* **38,** 36–45. ISSN: 1614-0001. https://doi.org/10.1027/1614-0001/a000220 (2017).

2. Austin, E. J., Manning, J. T., McInroy, K. & Mathews, E. A preliminary investigation of the associations between personality, cognitive ability and digit ratio. *Personality and Individual Differences* **33,** 1115–1124. https://doi.org/10.1016/S0191-8869(02)00002-8 (2002).

3. Bailey, A. A. & Hurd, P. L. Depression in men is associated with more feminine finger length ratios. *Personality and Individual Differences* **39,** 829–836. https://doi.org/10.1016/j.paid.2004.12.017 (2005).

4. Eichler, A. *et al.* Digit ratio (2D:4D) and behavioral symptoms in primary-school aged boys. *Early Human Development* **119,** 1–7. ISSN: 0378-3782. https://doi.org/10.1016/j.earlhumdev.2018.02.012 (2018).

5. Fink, B., Manning, J. T. & Neave, N. Second to fourth digit ratio and the 'big five' personality factors. *Personality and Individual Differences* **37,** 495–503. https://doi.org/10.1016/j.paid.2003.09.018 (2004).

6. Li, W. *et al.* Depressed female cynomolgus monkeys (macaca fascicularis) display a higher second-to-fourth (2D:4D) digit ratio. *Zoological Research* **40,** 219–225. ISSN: 2095-8137. https://doi.org/10.24272/j.issn.2095-8137.2019.022 (2019).

7. Lippa, R. A. Finger lengths, 2D:4D ratios, and their relation to gender-related personality traits and the big five. *Biological Psychology* **71,** 116–121. ISSN: 0301-0511. https://doi.org/10.1016/j.biopsycho.2005.02.004 (2006).

8. Lombardo, M. V. *et al.* Fetal programming effects of testosterone on the reward system and behavioral approach tendencies in humans. *Biological Psychiatry* **72,** 839–847. ISSN: 0006-3223. https://doi.org/10.1016/j.biopsych.2012.05.027 (2012).

9. Löwe, B., Kroenke, K. & Gräfe, K. Detecting and monitoring depression with a two-item questionnaire (PHQ-2). *Journal of Psychosomatic Research* **58,** 163–171. https://doi.org/10.1016/j.jpsychores.2004.09.006 (2005).

10. Manning, J. T. & Fink, B. Digit ratio (2D:4D), dominance, reproductive success, asymmetry, and sociosexuality in the BBC Internet Study. *American Journal of Human Biology* **20.** Publisher: Wiley Online Library, 451–461. https://doi.org/10.1002/ajhb.20767 (2008).

11. Manning, J. T. & Fink, B. Digit Ratio (2D:4D) and aggregate personality scores across nations: Data from the BBC internet study. *Personality and Individual Differences. Digit Ratio (2D:4D) and Individual Differences Research* **51,** 387–391. ISSN: 0191-8869. https://doi.org/10.1016/j.paid.2010.05.040 (2011).

12. Martin, S. M., Manning, J. T. & Dowrick, C. F. Fluctuating asymmetry, relative digit length, and depression in men. *Evolution and Human Behavior* **20,** 203–214. https://doi.org/10.1016/S1090-5138(99)00006-9 (1999).

13. Rapoza, K. A. Does life stress moderate/mediate the relationship between finger length ratio (2D4D), depression and physical health? *Personality and Individual Differences* **113,** 74–80. ISSN: 0191-8869. https://doi.org/10.1016/j.paid.2017.03.009 (2017).

14. Richter, D. & Schupp, J. The SOEP Innovation Sample (SOEP-IS). *Journal of Contextual Economics.* http://elibrary.duncker-humblot.com/zeitschriften/id/24/vol/135/iss/1623/art/7258/ (2015).

15. Sanwald, S. *et al.* Depression is associated with the absence of sex differences in the 2D:4D ratio of the right hand. *Frontiers in Psychiatry* **10.** ISSN: 1664-0640. https://doi.org/10.3389/fpsyt.2019.00483 (2019).

16. Sindermann, C. *et al.* The 2D:4D-ratio and neuroticism revisited: Empirical evidence from Germany and China. *Frontiers in Psychology* **7.** ISSN: 1664-1078. https://doi.org/10.3389/fpsyg.2016.00811 (2016).

17. Smedley, K. D., McKain, K. J. & McKain, D. N. 2D:4D digit ratio predicts depression severity for females but not for males. *Personality and Individual Differences* **70,** 136–139. https://doi.org/10.1016/j.paid.2014.06.039 (2014).

18. Vermeersch, H., T'Sjoen, G., Kaufman, J. M. & Vincke, J. 2D:4D, sex steroid hormones and human psychological sex differences. *Hormones and Behavior* **54,** 340–346. ISSN: 0018-506X. https://doi.org/10.1016/j.yhbeh.2008.02.017 (2008).

19. Voracek, M., Offenmüller, D. & Dressler, S. G. Sex differences in directional asymmetry of digit length and its effects on sex differences in digit ratio (2D:4D). *Percept Mot Skills* **107.** Publisher: SAGE Publications Inc, 576–586. ISSN: 0031-5125. https://doi.org/10.2466/pms.107.2.576-586 (Oct. 2008).

20. Walther, A. *et al.* Associations between digit ratio (2D4D), mood, and autonomic stress response in healthy men. *Psychophysiology* **56,** e13328. ISSN: 1469-8986. https://doi.org/10.1111/psyp.13328 (2019).

21. Williams, J. H. G., Greenhalgh, K. D. & Manning, J. T. Second to fourth finger ratio and possible precursors of developmental psychopathology in preschool children. *Early Human Development* **72,** 57–65. ISSN: 0378-3782. https://doi.org/10.1016/S0378-3782(03)00012-4 (2003).
